# Database development to maximise recruitment in a busy cardiac surgery department

**DOI:** 10.1186/1745-6215-14-S1-P59

**Published:** 2013-11-29

**Authors:** Lucy Dreyer, Katie Pike, Neil Smith, Lucy Culliford

**Affiliations:** 1 University of Bristol, Bristol, UK

## Introduction

The Bristol Clinical Trials and Evaluation Unit run multiple cardiac surgery trials in the Bristol Heart Institute (BHI), where approximately 1600 cardiac surgery operations take place annually. We will describe the operational challenges of managing the recruiting process across a range of studies and the database management solution we have developed to help us maximise recruitment.

## Challenges

1. Some trials only recruit from specific population sub-types.

2. Recruitment into RCTs and observational studies needs to be coordinated.

3. Theatre slot assignments are subject to constant change.

4. The NHS theatre list coordinators use a paper-based system.

5. A ‘Day of Surgery Admission’ policy means many patients need to be recruited at pre-surgery clinics.

## Database development

The database is used to capture details of all patients due to have surgery at the BHI, which enables us to systematically target potential patients for recruitment into a study. Limited patient details are entered onto the database and run through an algorithm to automatically assign the patient to the appropriate trial and/or observational study, based on trial priority and other eligibility criteria. Personalised invitation letters are automatically generated and sent with the Patient Information Leaflet as far in advance as possible of the surgery date. Automated e-mails are sent to the study team informing them of potential new patients and changes to existing theatre slots.

## Summary/conclusion

The system allows us to systematically identify and approach patients for participation in research, minimising bias and maximising potential recruitment from a large patient population.

